# In Situ Construction of Multi‐Functional Polymer Network Toward Durable Perovskite Solar Cells

**DOI:** 10.1002/advs.202503417

**Published:** 2025-04-30

**Authors:** Bingqian Zhang, Qiangqiang Zhao, Kun Gao, Xiaoxu Zhang, Caiyun Gao, Xiuhong Sun, Hongpei Ji, Xiaopeng Feng, Yaliang Han, Xiaofei Yan, Xiao Wang, Zhipeng Shao, Shuping Pang, Kezheng Chen, Guanglei Cui

**Affiliations:** ^1^ College of Materials Science and Engineering Qingdao University of Science and Technology Qingdao 266042 P. R. China; ^2^ State Key Laboratory of Photoelectric Conversion and Utilization of Solar Energy Qingdao New Energy Shandong Laboratory Qingdao Institute of Bioenergy and Bioprocess Technology Chinese Academy of Sciences Qingdao 266101 P. R. China; ^3^ Center of Materials Science and Optoelectronics Engineering University of Chinese Academy of Sciences Chinese Academy of Sciences Beijing 100049 P.R. China

**Keywords:** In situ polymerization, weak chemical bond network, perovskite solar cells and modules, long‐term stability

## Abstract

Enhancing the crystalline quality of perovskite thin films and stabilizing their internal grain boundaries are essential in guaranteeing the extended longevity of perovskite solar cells. Herein, an in situ polymerization strategy is presented to produce weak chemical bond networks in perovskite films. The introduction of acrylamide monomer into the perovskite precursor solution facilitates the rearrangement of [PbI_6_]^4−^ octahedra, resulting in a significant enhancement of the crystal quality of the perovskite films. With the presence of C═C bonds, the in situ polymerization of acrylamide at grain boundaries can form polymer networks, which can efficiently passivate the detrimental defects associated with grain boundaries. The perovskite solar cells with an impressive power conversion efficiency (PCE) of 26.05% (certified at 25.06%) are achieved, combined with highly improved operational stability with *T*_98_ = 2034 h. As expected, large‐area module based on this strategy achieved an impressive PCE of 23.02% with an active area of 14 cm^2^.

## Graphical abstract

Acrylamide monomers were introduced into the perovskite precursor solution to 3D polymer networks at grain boundaries for stabilizing grain boundaries. By constructing weak chemical bond interactions with perovskite films, the polymer networks effectively reduce defect density and simultaneously suppress the degradation of perovskites. Therefore, a champion efficiency of 26.05% was achieved with module efficiency of 23.02%.

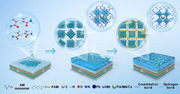

## 1 Introduction

Perovskite solar cells (PSCs) have achieved outstanding power conversion efficiency (PCE) exceeding 26%, positioning them as promising candidates for the next generation of photovoltaic technology.^[^ ^1^ ^]^ However, the commercialization of PSCs still faces significant challenges in terms of stability. Performance degradation commonly arises from defects at grain boundaries and interfaces, such as undercoordinated Pb^2+^ ions, cation or halide vacancies, interstitial defects, and other intrinsic point defects.^[^ ^2^ ^]^ This phenomenon is particularly noticeable when subjected to external pressures such as prolonged light exposure, increased humidity, and elevated temperatures.^[^ ^3^ ^]^ Conversely, the aforementioned conditions further accelerate defect formation, charge accumulation, ion migration, and non‐radiative recombination, leading to the structural deterioration of perovskite thin films and initiating subsequent chain reactions. Hence, minimizing defect density in both the perovskite bulk and interface is essential for ensuring the prolonged stability of PSCs.^[^ ^4^ ^]^

Nowadays, a series of molecules have been employed to alleviate recombination loss, thereby improving both PCE and stability of the devices.^[^ ^5^ ^]^ These molecules include small organic compounds,^[^ ^2^, ^6^ ^]^ polymers,^[^ ^7^ ^]^ and ammonium salts.^[^ ^8^ ^]^ Extensive research has predominantly focused on structural modification of small molecules, especially in relation to carbonyl and amide groups, through methods such as functional‐group tailoring and conformational design.^[^ ^2^, ^7^, ^9^ ^]^ It is worth noticing that these small organic molecules have a propensity to sublimate or diffuse, leading to the instability of surface and grain boundaries characterized by the emergence of pinholes and residual PbI_2_ over prolonged operation. This severely impacts the device's performance when subjected to heat, light, and humid conditions.^[^ ^10^ ^]^

Polymers play a crucial role in durably stabilizing interface structures and creating a physical barrier to prevent the volatilization of organic cations, as well as the intrusion of moisture and oxygen. However, challenges arise when incorporating polymers into the precursor solution. Owing to the poor solubility and the steric hindrance, polymer additives can hinder nucleation and bring unfavorable growth processes of perovskite, resulting in inherent resistance to crystallization or even precipitation formation. This interference ultimately hinders the production of high‐quality perovskite films.^[^ ^11^ ^]^ Furthermore, certain polymers such as polyacrylonitrile^[^ ^12^ ^]^ and poly(methyl methacrylate‐*co*‐acrylamide)^[^ ^2a^ ^]^ theoretically have effective passivation properties, their limited solubility in polar aprotic solvents like dimethyl sulfoxide and *N*,*N*‐dimethylformamide poses a barrier to their extensive application in the modification of internal grain boundaries.^[^ ^13^ ^]^ Monomer molecules could not impede the growth of perovskite crystals; moreover, they are capable of undergoing spontaneous polymerization to ensure long‐term stability.^[^ ^14^ ^]^ This motivates us to address the challenges associated with small molecules and polymer additives through the implementation of in situ polymerization by multifunctional small molecules.

In this study, we introduce an in situ polymerization strategy that generates weak chemical bond networks (WCBN) within perovskite films. By incorporating acrylamide (AM) monomers into the perovskite precursor solution, we achieve significant improvements in the crystalline quality of the thin films. During thermal annealing, polyacrylamide (PAM) networks form in situ, establishing a stable architecture of WCBN. Such network structure can stabilize grain boundaries and improve the long‐term stability of the devices. The as‐fabricated PAM‐based PSCs exhibited a PCE of 26.05% (certified at 25.06%), combined with excellent operational and storage stability.

## 2 Results and Discussion

**Figure 1:**
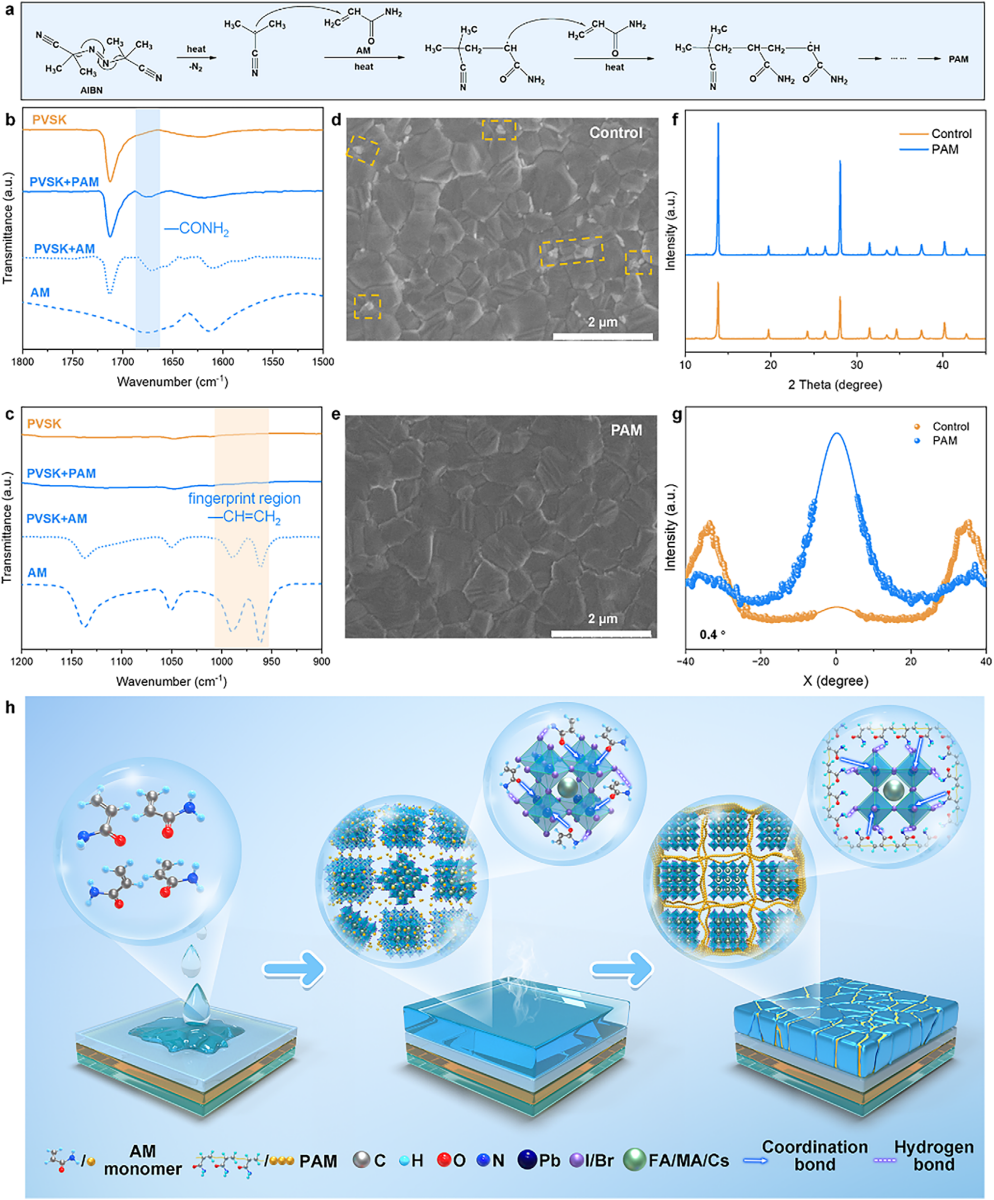
Schematic illustration of in situ polymerization of acrylamide in perovskite film. a) In situ polymerization reaction of PAM. b,c) FTIR spectra of acrylamide, perovskite film, and perovskite film with PAM, respectively. d,e) Top‐view SEM images, f) XRD patterns, and g) Azimuthal tube integrals of the (100) Debye rings of the control and PAM‐treated film, respectively. (h) Diagram of the weak chemical bond network of PAM with flexible polymer chains.

Owing to the polymerizable characteristics of acrylamide, we incorporated it into the perovskite precursor solution as an additive during the spin‐coating process. Subsequently, in situ polymerization occurred during the annealing process of the perovskite film. Briefly, with the absence of 2,2′‐azobis(2‐methylpropionitrile) (AIBN) initiator, PAM is obtained through free radical polymerization reaction in the annealing process of perovskite film (**Figure** **1**a; Figure S1, Supporting Information).^[^ ^15^ ^]^ We have also selected some molecules with typical functional groups (carboxyl group, cyano group, and ester group) and compared the in situ polymerization effects of AM, acrylic acid (AA), acrylonitrile (AN), and methyl acrylate (MA) on the X‐ray diffraction (XRD) peaks of perovskite films as shown in Figure S2 (Supporting Information). With the introduction of these molecules, PAM‐based perovskite films have the best crystallinity, and we will discuss its crystallization process later.

To examine the polymerization of AM and confirm the formation of PAM within the perovskite film after annealing, Fourier‐transform infrared spectroscopy (FTIR) analysis was conducted. As can be seen in Figure 1b,c, there exists an obvious bulge at ≈1670 cm^−1^ with a frequency shift of 5 cm^−1^, which refers to the C═O stretching vibration absorption peak of the amide group. A 5 cm^−1^ shift shows the coordination bond (C═O→Pb) between polymer and perovskite film. Moreover, the fingerprint region of the ─CH═CH_2_ peak at 950–1000 cm^−1^ in AM has disappeared, demonstrating the successful polymerization from AM to PAM. To further verify the WCBN mechanism, X‐ray photoelectron spectroscopy (XPS) was conducted as shown in Figures S3, S4 (Supporting Information). The core level spectra of Pb 4f and I 3d in the perovskite exhibit a shift toward lower binding energy (0.26 and 0.24 eV, respectively), which refers to the coordination bond (C═O→Pb) and hydrogen bond (N─H…I), respectively. Additionally, to gain insights into the optical properties and interactions within the perovskite film influenced by PAM, UV–vis absorption and steady‐state photoluminescence (PL) spectra were analyzed (Figure S5, Supporting Information). There is no change in the band gap, while the PL intensity of the PAM‐treated perovskite film increased two times that of the control film. The increased PL intensity and the 2 nm blue shift of the PL peak both illustrate the reduction of defects, which will further enhance the device's performance. Moreover, time‐resolved PL spectra (Figure S6, Supporting Information) were performed to study carrier dynamics of perovskite films. The averaged carrier lifetime increased from 288 to 390 ns, indicating that PAM is effective in suppressing unfavored non‐radiative recombination by the construction of WCBN.

Scanning electron microscopy (SEM), Transmission electron microscopy (TEM), XRD, and grazing incident wide‐angle X‐ray scattering (GIWAXS) measurements were also conducted to further understand the insight of the perovskite film crystallization. As presented in Figure 1d,e, the PAM‐containing film exhibits similar grain size and clearer grain boundaries than the control film.^[^ ^16^ ^]^ For the cross‐sectional images shown in Figure S7a,b (Supporting Information), we find that there exist some double‐layer grains in some regions of the control film, leading to inferior carrier transportation. Besides, the perovskite film of TEM images with PAM treated shows that there exists polymer at grain boundaries of polycrystalline perovskite, which further demonstrates the distribution of PAM and the construction of WCBN (Figure S7c,d, Supporting Information). The diffraction peak at 14.2 ° in the XRD pattern is attributed to the (100) crystal plane of the perovskite structure. The enhanced intensity of this peak indicates improved crystallinity of the perovskite films, as shown in Figure 1f and Figure S8 (Supporting Information). The perovskite films without and with PAM both show characteristic diffraction rings at q_r_ = 1.00 and 1.40 Å^−1^ which refers to the (100) and (110) planes of perovskite polycrystals, respectively. The signal of PbI_2_ at q_r_ = 0.90 Å^−1^ is reduced after the introduction of PAM, indicating that this in situ network construction can restrain the defects both at the surface and in the bulk of the perovskite. Besides, azimuthal tube integrals of the (100) Debye rings extracted from 2D GIWAX data are depicted in Figure 1g.^[^ ^17^ ^]^ There exists an obvious peak at ≈0°, which means the regulation of vertical orientated perovskite by PAM. To gain more insights into the remarkably enhanced film quality, we adopted in situ PL spectra to understand the role of PAM during film formation. The nucleation and crystallization process become faster after the introduction of AM into perovskite precursor solution (Figure S9, Supporting Information), showing that the monomer has reduced the energy barrier of [PbI_6_]^4−^ octahedron and further enhanced the quality of perovskite crystallization. These results confirm that WCBN is successfully constructed due to the interaction between the amide group and perovskite film.^[^ ^18^ ^]^ Consequently, the above‐mentioned results can be well corroborated by each other, and the possible mechanism process is depicted in Figure 1h.

To study the effect of WCBN between PAM and perovskite films, we conducted theoretical calculations based on density functional theory (DFT). As represented in Figure S10 (Supporting Information), the calculated electrostatic potential distribution of acrylamide pentamer revealed strong interactions between the polymer model and lead/iodine, which is because of the multi‐active sites of pentamer compared with monomer. Notably, the dipole moment of pentamer (5.65 D) is much higher than that of monomer (3.53 D), further demonstrating the stronger interaction between PAM and perovskite film. In addition, the flexible chain of PAM polymer contributes to the in situ construction of WCBN, and allows for more adaptable interactions with lead/iodine in perovskite film at grain interface. Thus, we calculated the theoretical interaction between PbI_2_ and monomer/pentamer (Figure S11, Supporting Information). Compared with the monomer, pentamer has a stronger interaction with PbI_2_ and increases from −0.125 to −0.665 eV. Moreover, the binding energy between perovskite and pentamer is −0.180 eV, which is 3.5 times more than the monomer (Figure S12, Supporting Information). The enhanced interaction and binding energy demonstrate the superior effect of PAM.

**Figure 2:**
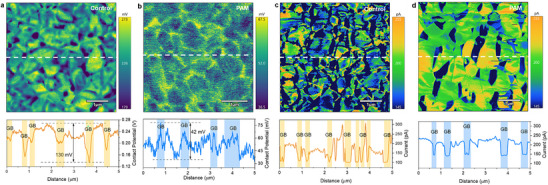
Electronic properties of perovskite films. KPFM of contact potential differences and line profiles of the contact potential differences for a) control film and b) PAM‐based film, respectively. c‐AFM of contact potential differences and line profiles of the current differences for c) control film and d) PAM‐based film, respectively.

Subsequently, the optoelectronic properties of perovskite films without/with PAM were also investigated. The atomic force microscopy (AFM) results in Figure S13 (Supporting Information) show that surface morphology has changed significantly before and after in situ polymerization, with the surface roughness (RMS) changed from 42.15 to 30.94 nm. In addition, nano‐IR was conducted to investigate the distribution of PAM. As can be seen in Figure S14 (Supporting Information), the signal of the amide group distributes on the surface of the perovskite film. These results imply that PAM polymer is distributed at grain boundaries of the polycrystalline perovskite, and thus improves the surface uniformity. Also, Kelvin probe force microscopy (KPFM) is carried out to study the contact potential difference (CPD) of the electrical properties for perovskite films without/with PAM. The results in **Figure** **2**a,b show that the CPD declines remarkably with the introduction of PAM, which means the work function of the film shifts downward, thus achieving a better energy level alignment. The difference of CPD between GBs and adjacent grains of PAM is significantly reduced to 42 mV, compared with that of the control film (130 mV). This flattened potential fluctuation demonstrates the distribution of PAM at GBs and thus further inhibits the unfavored carrier recombination.^[^ ^19^ ^]^ Besides, conductive AFM (c‐AFM) data in Figure 2c,d further revealed that the treatment of PAM modified the electric properties, with the improved homogeneity in current distribution for spatially averaged current increased from 161 pA for the control to 193 pA for the PAM film, respectively. The currents at the intragrain of perovskite film with PAM are higher than those without PAM modification, which means the polycrystalline perovskite regulation of vertical orientation with PAM is of great benefit to charge transportation.

**Figure 3:**
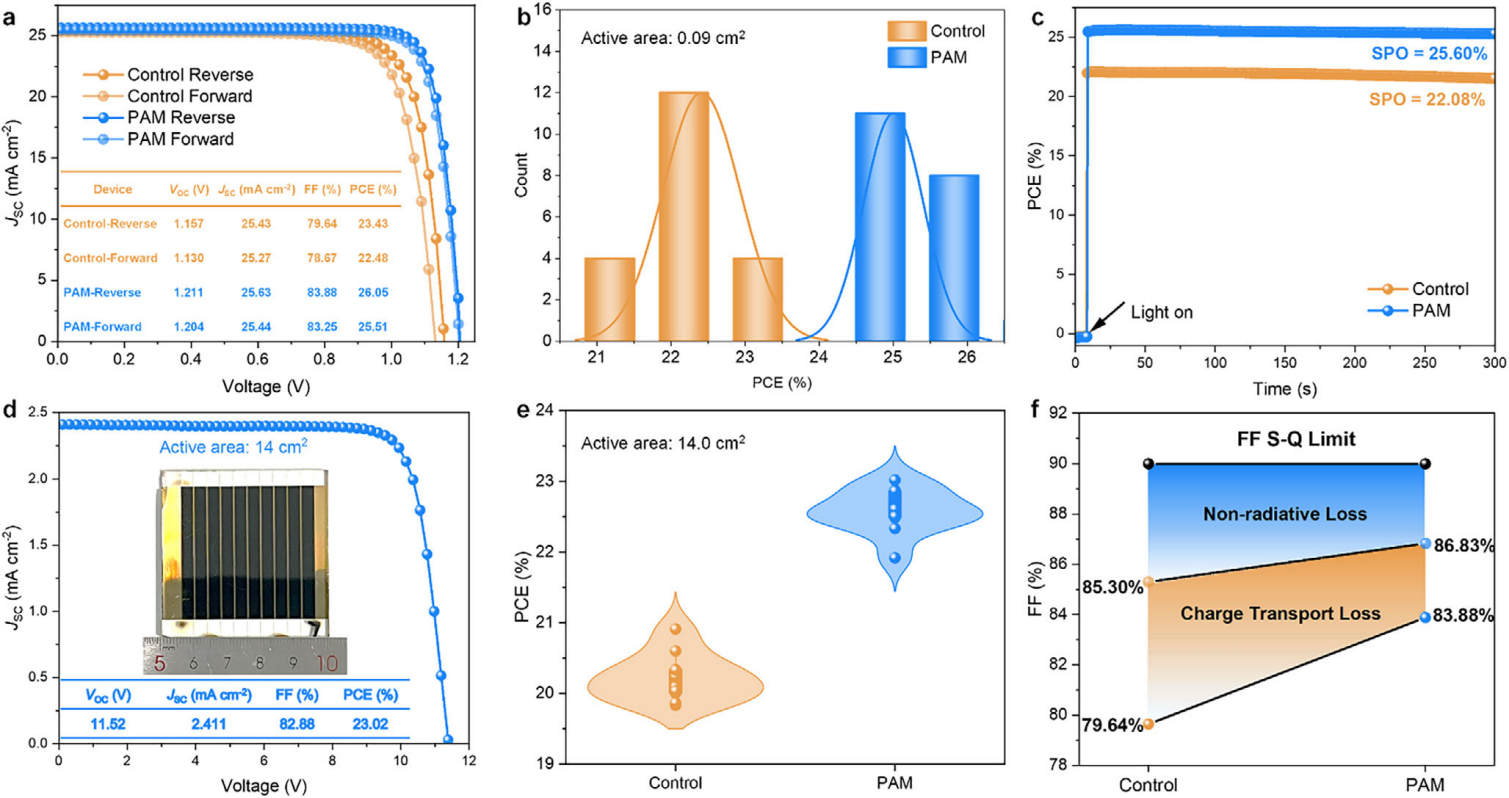
Performance of control and PAM‐based PSCs and modules. a) *J*–*V* curves of the control and champion devices. b) Histogram of the PCE distribution with an active area of 0.09 cm^2^. c) SPO curves of the control and champion devices. d) *J*–*V* curve of the champion perovskite solar module with an active area of 14 cm^2^. e) Histogram of the PCE distribution for perovskite solar modules without and with PAM. f) The FF S–Q limit of the control and champion devices consists of charge‐transport loss and non‐radiative loss, respectively.

To investigate the effect of WCBN on the device performance, we fabricated PSCs based on the architecture of FTO/c‐TiO_2_/SnO_2_/perovskite/spiro‐OMeTAD/Au. With the introduction of PAM, the target device shows great improvement in all photovoltaic parameters, especially the *V*_OC_ and FF, and we obtained the best concentration of AM additive for 8 mg mL^−1^ (Figure S15 and Table S1, Supporting Information). As shown in **Figure** **3**a–c, a champion device is achieved with PCE of 26.05% (stabilized at 25.60%), *V*_OC_ of 1.211 V, *J*_SC_ of 25.63 mA cm^−2^, and FF of 83.88%, while the control device only has PCE of 23.43% (stabilized at 22.08%), *V*_OC_ of 1.157 V, *J*_SC_ of 25.43 mA cm^−2^, and FF of 79.64%. In addition, the certified PCE of the champion device is 25.06% as presented in Figure S16 (Supporting Information). The slight increase in *J*_SC_ by 0.2 mA cm^−2^ obtained from the *J*‐*V* measurements is consistent with the EQE‐*J*_SC_ curves (Figure S17, Supporting Information) and c‐AFM results (Figure 2c,d), probably due to the distribution of PAM at the grain boundaries and surface of polycrystalline perovskite. Notably, the hysteresis index of the champion device is 2.1%, compared to 4.0% of the control device (Table S2, Supporting Information), indicating the possible effect of WCBN on reducing the non‐radiation recombination and ion migration. Figure 3b and Figure S18 (Supporting Information) exhibit the average PCE histogram of 20 devices of the control and target devices. The PAM‐based device is increased by 11.6%, from 22.41 ± 0.52% to 25.00 ± 0.43%, and the average *V*_OC_ is impressively enhanced from 1.120 ± 0.018 V to 1.186 ± 0.010 V.

Furthermore, the ideal factor calculated by the fitting curves in Figure S19 (Supporting Information) of the champion device with PAM treatment is reduced to 1.40 compared with 1.55 of the control device, demonstrating an ameliorative charge recombination process. *V*_OC_ is inversely proportional to leakage current, which means that the improvement of *V*_OC_ should be accompanied by the decrease of leakage current. Thus, the dark *J*–*V* curves were measured as shown in Figure S20 (Supporting Information). It implies that the leakage current of PAM‐based device is reduced by two orders of magnitude than the control device. Besides, the enhanced transient photovoltage (TPV) result (Figure S21, Supporting Information) also proves the improvement of *V*_OC_. The effect of PAM on the charge‐transport behavior was further evaluated by the space charge limited current (SCLC) and electrochemical impedance spectroscopy (EIS) measurements. As shown in Figure S22 (Supporting Information), both hole and electron mobility of PAM‐based devices are slightly increased compared to the control devices, demonstrating that the in situ polymerization of PAM will not reduce the charge transport properties of the devices. Additionally, the Nyquist plot of the PAM‐based device exhibits a small charge‐transport resistance (*R*_ct_) and a bigger recombination resistance (*R*_rec_) compared with the control device. The result indicates that, with the construction of WCBN, it can make more efficient charge transport inside the device, which is consistent with the TPV result. To find out which defects were reduced and trap density changes of devices, we conducted thermal admittance spectroscopy. The trap density of state (tDOS) profiles in Figure S23 (Supporting Information) showed that both the density of negatively charged iodine interstitials (*I*_i_^−^) and positively charged iodine interstitials (*I*_i_^+^) are reduced by the introduction of PAM. ^[^ ^20^ ^]^ It is also demonstrated that shallow‐level defects effectively passivated from the magnitude of ≈10^17^ to ≈10^16^.

The compatibility of this in situ polymerization strategy with scaled‐up processes was further evaluated by fabricating perovskite solar modules based on FTO substrates (50 × 50 mm). Detailed fabrication information of perovskite solar modules is provided in Supporting Information. Briefly, a module consists of 10 sub‐cells connected in series, and each sub‐cell is divided into equal sizes with a width of 4 mm by laser scribing of P1, P2, and P3 lines (Figures S24–S26, Supporting Information). The champion module was obtained for an active area of 14 cm^2^ and a geometrical fill factor of 87.5%, respectively, with a remarkable PCE of 23.02%. Figure 3e shows the PCE histogram of 10 modules (more detailed parameters are listed in Figure S27, Supporting Information), with the average PCE of PAM‐based modules increased from 20.22 ± 0.34% to 22.64 ± 0.23%. It is worth noticing that the average *V*_OC_ and FF are also improved, from 11.04 ± 0.190 to 11.49 ± 0.061 V and 77.16 ± 0.96% to 81.99 ± 0.44%, respectively. These results further illustrate that the scaled‐up modules still have good compatibility and repeatability with the WCBN interaction by the introduction of PAM.

To reveal the mechanism of performance enhancement by the addition of AM, we conducted the Shockley–Queisser (S–Q) limit of FF analysis.^[^ ^21^ ^]^ The FF loss primarily involves trap‐assisted non‐radiative loss and charge transport loss. As depicted in Figure 3f, the calculated FF_max_ values are 85.30% and 86.83% for control and PAM‐based devices, respectively. It is obvious that the FF loss in the PAM‐treated device is lower than in the control device owing to the suppression of trap‐assisted recombination. The charge transport loss in FF is also decreased by the treatment of PAM thanks to the optimized carrier extraction and transportation in the device. Moreover, the S‐Q limit of *V*_OC_ analysis also shows that, with the in situ construction of coordination/hydrogen bonding, the *V*_OC_ boosts to over 95% of the S‐Q limit (Figure S28, Supporting Information). These results demonstrate that the improved FF is mainly contributed by the molecular engineering of PAM, which agrees well with the results in the perovskite film characterization. Thus, the presence of WCBN will not only suppress the non‐radiative recombination but also raise charge transport in the device.

Since the PCE of PSCs are closely related to the energy loss from transmission, non‐absorbing, thermalization, and carrier dynamics (containing non‐unity internal quantum efficiency (IQE) and FF, and overall *V*_OC_ loss) that are inevitable during the energy conversion process, we have quantitatively analyzed the energy loss from each channel for the control and PAM‐based devices based on the reported references.^[^ ^22^ ^]^ More detailed information of calculation is discussed in Supporting Information. The results of the control and PAM‐treated devices are shown in Figure S29 (Supporting Information), and energy loss values explicitly summarize the comparison of those devices in Table S3 (Supporting Information). It is presented that the major energy loss of the device comes from the transmission with the same value of 37.0 mW cm^−2^, which is due to the bandgap of the absorber layer has no change. Besides, the energy loss of PAM‐based devices caused by the insufficient light absorbing loss (*E*_abs_) and IQE loss (*E*_IQE_) are also alleviated from 13.8 mW cm^−2^ (control) to 12.4 mW cm^−2^ (PAM‐treated), attributing to the greater EQE response for the PAM‐treated device. From Equation S5 (Supporting Information) we can see that, *E*_therm_ depends on the *E*_g_ of the solar cell system and EQE response. Here, the *E*_g_ of these two devices are identical, i.e., the difference of *E*_therm_ for both is only affected by EQE spectra. As a result, *E*_therm_ of PAM‐based device is reduced from 16.0 mW cm^−2^ (control) to 15.0 mW cm^−2^. It is worth noting that both $E_{V_{\mathrm{oc}}}$ and *E*_FF_ of PAM‐treated device shows remarkable reduction from 7.15 mW cm^−2^ (control) to 6.56 mW cm^−2^ and from 4.48 mW cm^−2^ (control) to 4.02 mW cm^−2^, respectively, which attributes to the better optoelectrical performance of the device. Based on the above‐mentioned analyses, we obtain the total conversion energy with the values of 17.5 mW cm^−2^ for the control and 20.9 mW cm^−2^ for the PAM‐treated device, respectively.

**Figure 4:**
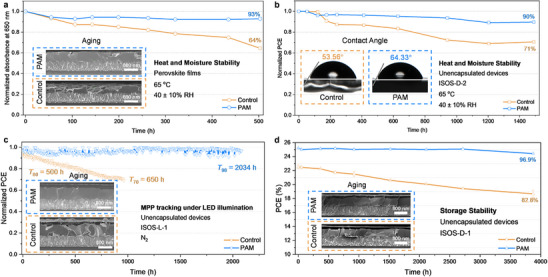
Enhanced long‐term stability of perovskite films and devices. a) Normalized UV–vis absorption spectra at 650 nm with different aging times. (Inset: Cross‐sectional SEM images of perovskite films without and with PAM treatment after aging for 503 h.) b) ISOS‐D‐2 stability of the unencapsulated devices under 40 ± 10% RH at 65 °C. (Inset: The contact angle of pristine films without and with PAM treatment.) c) ISOS‐L‐1 stability of unencapsulated devices measured at room temperature and nitrogen atmosphere. (Inset: Cross‐sectional SEM images of devices without and with PAM treatment after continuous illumination.) d) ISOS‐D‐1 stability of the unencapsulated devices measured at ambient conditions. (Inset: Cross‐sectional SEM images of devices without and with PAM treatment after storage for 3874 h.).

It has been proved that WCBN takes an active role in device efficiency; device stability is of great importance as well. To address this issue, long‐term stability tests under various conditions were taken to give further investigation and comprehension. We have first investigated the degradation process of perovskite films without and with PAM treatment under 40 ± 10% RH at 65 °C continuous heating. As can be seen in **Figures** **4**a and S30 (Supporting Information), the control film drops to 64% of its initial absorption, while PAM‐treated film remains 93% after aging for over 500 h. The insert of cross‐sectional SEM images in Figure 4a directly shows the collapse of perovskite structures. There are only fewer pinholes and PbI_2_ on PAM‐treated films, compared to the control film with severely collapsed structures. The XRD patterns of these aged films further proved it (Figure S31, Supporting Information). For the control film, the (100) crystal plane of perovskite nearly disappears after 500 h aging and exhibits a strong PbI_2_ peak. On the contrary, the (100) crystal plane of PAM‐treated film maintains a strong peak, which is weaker than PbI_2_ peak. The color change of optical photographs before and after aging (Figure S32, Supporting Information) and top‐view SEM images (Figure S33, Supporting Information) also demonstrate the effect of PAM on the enhancement of heat and moisture stability of the perovskite films. To further quantitatively analyze the aging process of perovskite film, we then carried out energy dispersive spectrometer measurements. The I/Pb ratio of the pristine films is 2.98 for the control one and 3.08 for the PAM‐treated film, respectively, which shows the hydrogen bonding between PAM and perovskite for immobilizing the iodine (Tables S4 and S5, Supporting Information). After over 500 h degradation process, the I/Pb ratio of the aged films changes to 2.24 for the control one and 2.50 for the PAM‐treated film, respectively. The more ideal I/Pb ratio of the target film indicates the protection with WCBN and the reduction of ion migration.

Then we evaluated the stability of PSCs based on the International Summit on Organic Photovoltaic Stability (ISOS) protocols reported previously.^[^ ^23^ ^]^ Figure 4b shows the heat and moisture stability (ISOS‐D‐2) of unencapsulated devices under 40 ± 10% RH at 65 °C. The devices based on PAM retain over 90% of their initial PCE after heating continuously for 1488 h, while the control devices decline to 71%. In addition, the results of contact angle measurement for the perovskite film without and with PAM treatment also help to illustrate the improved stability of PSCs. As shown in the insert of Figure 4b, the contact angle of PAM‐treated film is 64.33 °, compared to the 53.56 ° for the control film. The increase in the contact angle indicates the existence of a modified layer on the surface of the perovskite film, which can protect the perovskite layer without moisture and prevent the volatilization of perovskite components. Under the light‐soaking stability (ISOS‐L‐1) of unencapsulated devices under N_2_ with maximum power‐point (MPP) tracking in Figure 4c, the PCE of the PAM‐based devices keeps 95% of the initial value after 1000 h, whereas the control devices drop to 80% of their initial PCE after 500 h and show over 30% degradation for 960 h. The insert of Figure 4c shows the cross‐section SEM images after MPP tracking. The PAM‐based devices display distinct perovskite grains without pinholes and PbI_2_, while the perovskite structures of control devices collapse with obvious generation of PbI_2_. Finally, the storage stability (ISOS‐D‐1) of PSCs is investigated in Figure 4d. The PCE of PAM‐based devices only decreases by 3.1% of their initial values, whereas the control devices fall to 82.8% after 3874 h. The champion perovskite solar module exhibits great storage stability (ISOS‐D‐1) with the maintenance of 97.8% of its initial value (Figure S34, Supporting Information) for over 2173 h. The enhanced stability under different conditions after the introduction of PAM to form WCBN can be attributed to the excellent stability against moisture as well as their improved charge extraction, carrier transportation, and reduced defect density of metal‐cation and halide‐anion‐related defects.

## 3 Conclusion

To summarize, the introduction of AM monomers into the perovskite precursor solution can improve the crystallinity of perovskite films. The polymerized 3D networks form WCBN at grain boundaries, which is conducive to reducing the defect density and enhancing damp heat stability. The in situ polymerization approach utilized in PSCs achieved an efficiency of 26.05%. Moreover, it demonstrated excellent stability with 98% retention after 2034 h for the ISOS‐L‐1 stability test, and with 97% over 3800 h for the ISOS‐D‐1 stability test, respectively. The photoelectric properties and analysis of defect states confirm the significant role of PAM in enhancing charge transfer and reducing defect states. Energy loss calculation quantifies the lower device losses in transport and non‐radiative recombination. The as‐obtained perovskite solar modules also achieve an excellent PCE of 23.02% with superior long‐term stability. The findings of this study suggest that the in situ polymerization passivation molecules have a great potential to simultaneously enhance the efficiency and stability of PSCs.

## Conflict of Interest

The authors declare no conflict of interest.

## Supporting information



Supporting Information

## Data Availability

The data that support the findings of this study are available from the corresponding author upon reasonable request.
