# Supplementary material for: In Situ Construction of Multi‐Functional Polymer Network Toward Durable Perovskite Solar Cells
Source: Adv Sci (Weinh). 2025 Apr 30;12(27):2503417. doi: 10.1002/advs.202503417 (PMC12279194; doi:10.1002/advs.202503417)
Supplement: Supplementary file 1 — Supporting Information [file ADVS-12-2503417-s001.docx]

Supporting Information
©Wiley-VCH 2022
69451 Weinheim, Germany

Table of Contents

**Experimental Procedures**  **4**

Materials 4

Film fabrication 4

Device fabrication 4

Perovskite module fabrication 4

Materials characterization 4

Device Testing 5

Trap density of state (tDOS) measurements 5

S-Q limit of *V*_OC_ and FF analysis 5

Energy loss analysis 5

**Results and Discussion**  **6**

Figure S1. Schematic diagram of in-situ polymerization reaction 6

Figure S2. XRD patterns of the control, PAM-, PAA-, PAN-, and PMA-treated perovskite films. 6

Figure S3. XPS data of the control and PAM-treated perovskite films 6

Figure S4. Pb 4f and I 3d XPS spectra of the control and PAM-based films, respectively 6

Figure S5. (a) UV-vis absorption and (b) steady-state PL spectra of the control and PAM-based perovskite films 7

Figure S6. Time-resolved PL spectra of the control and PAM-based perovskite films 7

Figure S7. Cross-sectional SEM and TEM images of (a, c) control and (b, d) PAM-based perovskite films 7

Figure S8. GIWAXS patterns of (a) control and (b) PAM-based perovskite films 7

Figure S9. In-situ PL spectra (a) without and (b) with AM monomer... 8

Figure S10. Electrostatic potential profiles of monomer and pentamer... 8

Figure S11. Theoretical interaction between PbI_2_ and monomer/pentamer... 8

Figure S12. Binding energy between perovskite and monomer/pentamer... 9

Figure S13. AFM morphology of (a) control and (b) PAM film with the scan size of 5 × 5 µm... 9

Figure S14. (a, c) AFM morphology images and (b, d) Nano-IR images of control film and PAM-treated film... 9

Figure S15. *J-V* curves of PAM-based solar cells with different concentrations of AM additive... 10

Figure S16. Certified efficiency with a designed area of 0.08713 cm2, confirmed by the Photovoltaic and Wind Power Systems Quality Test Center, IEE, Chinese Academy of Sciences, Beijing, P. R. China... 10

Figure S17. EQE-*J*_SC_ curves of control and PAM-based solar cells... 10

Figure S18. The boxplots of photovoltaic performance for control and PAM-based solar cells... 11

Figure S19. *V*_OC_ response under different light intensities... 11

Figure S20. The dark current-voltage curves of control and PAM-based solar cells... 11

Figure S21. TPV curves of control and PAM-based solar cells... 11

Figure S22. Electrical characterization of the control and PAM-based solar cells measured in dark conditions.... 12

Figure S23. tDOS of the control and PAM-based solar cells... 12

Figure S24. Schematic illustration of laser scribing by P1, P2, and P3 lines for PSC modules... 12

Figure S25. (a) Schematic illustration and (b) SEM top-view image of the perovskite solar module showing the P1-P2-P3 parameters... 13

Figure S26. Front-view of schematic diagram for perovskite solar module structure... 13

Figure S27. The boxplots of photovoltaic performance for the control and PAM-based perovskite solar modules... 13

Figure S28. S-Q limit of *V*_OC_ analysis... 14

Figure S29. Detailed total energy loss analysis for the optimized: Control (a) and champion (b) device with the illumination of AM 1.5 G, 100 mW cm^-2^. (c) The corresponding Trans. Loss, Abs. and IQE Loss, *V*_OC_ loss, and FF loss... 14

Figure S30. UV-vis absorption spectra with different heating time of (a) control and (b) PAM-treated perovskite film... 14

Figure S31. XRD patterns of the control and PAM-treated perovskite film after aging for 503 h... 14

Figure S32. Photo images of perovskite films for (a) control and (b) with PAM before and after aging... 15

Figure S33. Top-view SEM images for control and PAM-based films after aging... 15

Figure S34. Storage stability for the perovskite solar module... 15

Table S1. Photovoltaic performance of PAM-based solar cells at different concentrations of AM 15

Table S2. Photovoltaic performance of control and PAM-based solar cells. 15

Table S3. The analysis parameters of the total energy loss for the control and PAM-based solar cells. 16

Table S4. EDS elements analysis for the pristine films without and with PAM. 16

Table S5. EDS elements analysis for the aged films without and with PAM. 16

**Author Contributions**  **16**

**References 17**

Experimental Procedures

*Materials:* Tin (Ⅳ) chloride, acrylic acid (AA), acrylonitrile (AN), methyl acrylate (MA), and acrylamide (AM) were purchased from Macklin. Formamidinium iodide (FAI), lead iodide (PbI_2_) was purchased from Advanced Election Technology CO., Ltd. 2,2'-Azobis(isobutyronitrile) (AIBN) and 4-*tert*-butylpyridine (*t*BP) were purchased from TCI. Methylammonium iodide (MAI), methylammonium chloride (MACl), methylammonium Lead Bromide (MAPbBr_3_) and cesium iodide (CsI) were purchased from Xi′an Yuri Solar Co., Ltd. 2,2″,7,7″-tetrakis[*N*,*N*-di(4-methoxyphenyl)amino]-9,9′-spirobifluorene (spiro-OMeTAD) was purchased from Borun New Material Technology Co., Ltd. Dimethyl sulfoxide (DMSO), *N*,*N*-dimethylformamide (DMF), anisole, chlorobenzene (CB) and lithium bis(trifluoro-methanesul-fonyl)imide (Li-TFSI) were purchased from Sigma Aldrich. Acetonitrile (ACN) and isopropanol (IPA) were purchased from Aladdin. High-purity gold was purchased from ZhongNuo Advanced Material (Beijing) Technology Co., Ltd. All materials were used as received without further purification except for recrystallization treatment of AIBN.

*Film fabrication:* FTO-coated glass (7 Ω sq^–1^) was cleaned via sequential sonication (30 min for each) with detergent in water for once, deionized water for twice, ethanol for once, and isopropanol for once (Notably, the detergent, ethanol, and isopropanol used here were all purchased from Greagent, Shanghai Titan Scientific Co., Ltd). The perovskite solution ((FA_0.95_Cs_0.05_)PbI_3_)_0.975_(MAPbBr_3_)_0.025_) was prepared by mixing 705.3 mg of PbI_2_, 228.8 mg of FAI, 18.2 mg of CsI, 33.67 mg of MACl and 18.2 mg of MAPbBr_3_ in mixed solvent of 890 µL DMF and 110 µL DMSO. Then the as-obtained solution was spin-coated on the FTO in a two-step at 1000 rpm (500 rpm ramp) and 4000 rpm (1000 rpm ramp) for 10 s and 30 s, respectively, in N_2_ glovebox. 300 μL anti-solvent anisole was drop-casted quickly in the center of FTO at the last 20 s of the second step. These films were transferred onto a hotplate in ambient air with relative humidity (RH)< 30% and heated at 100 °C for 40 min.

For PAM films, adding a range of AM (2 mg mL^-1^, 4 mg mL^-1^, 6 mg mL^-1^, and 8 mg mL^-1^, 10 mg mL^-1^, respectively) and 0.5 mg AIBN into the precursor solution. The perovskite layer was prepared as mentioned above.

*Device fabrication:* FTO-coated glass (7 Ω sq^–1^) was cleaned via sequential sonication (30 min for each) with detergent in water for once, deionized water for twice, and ethanol for once. The compact TiO_2_ layer was deposited by atomic layer deposition (ALD) and annealed in ambient air at 500 °C for 30 min. Then the SnO_2_-Cl layer was deposited by spin-coating at 3,000 rpm for 30 s and annealed in ambient air at 180 °C for 30 min. Specifically, SnO_2_ precursor solution was obtained by mixing the anhydrous SnCl_4_ solution with deionized water in a volume ratio of 1:75, and then the solution was aged at 25 °C for two weeks to obtain a white colloidal solution. The perovskite layer was prepared as mentioned above. The spiro-OMeTAD solution was prepared by dissolving 72.3 mg of Spiro-OMeTAD in 1 mL of chlorobenzene, and 46.3 µL of the mixed solution of 1.645 mL of *t*BP and 1 mL of Li-TSFI solution (520 mg of Li-TSFI in 1 mL of ACN were added. 40 µL of Spiro-OMeTAD solution was deposited on perovskite films by spin-coating (3000 rpm, 30 s). Finally, 80 nm thick Au electrodes were thermally evaporated under vacuum (< 4×10^-4^ Pa) to complete the device fabrication and ageing devices for 24 h in a drying air box.

*Perovskite module fabrication:* FTO-coated glass (7 Ω sq^–1^) with 50 × 50 mm was cleaned via sequential sonication (30 min for each) with detergent in water for once, deionized water for twice, ethanol for once, and isopropanol for once. Then, P1 lines with 30 μm etching process were patterned by a 1064 nm laser (Han' laser), with the laser power ratio, laser duty cycle, and laser frequency were 30%, 5%, and 50 kHz, respectively. The compact TiO_2_ layer was deposited by atomic layer deposition (ALD) and annealed in ambient air at 500 °C for 30 min. Next, the SnO_2_-Cl, perovskite, and the spiro-OMeTAD layers were prepared with the same procedure as device fabrication. Note that the volume of anti-solvent anisole was 600 μL during the perovskite layer process. Later, the P2 lines with 250 μm etching process were patterned by a 532 nm laser, with a laser power ratio of 65%, a laser duty cycle of 5%, and a laser frequency of 100 kHz, respectively. 80 nm thick Au electrodes were then thermally evaporated under a vacuum to complete the fabrication of the modules. Finally, 55-μm-wide P3 lines were etched using the same laser as P2 with a laser power ratio of 50%, a laser duty cycle of 5%, and a laser frequency of 100 kHz, respectively. P4 is an etching procedure for cleaning the edge of the modules, the laser used in P4 is the same as P1 with a laser power ratio of 40%, a laser duty cycle of 10%, and a laser frequency of 100 kHz. The geometric fill factor (GFF) is 87.5% for the perovskite module.

*Materials characterization*: XRD patterns were measured by a Bruker-AXS Micro diffractometer (D8 ADVANCE) with Cu K_α_ radiation (1.5406 Å). The optical absorbance spectra were measured by UV-vis/NIR spectrophotometer (U-4100, Hitachi). Steady Photoluminescence (PL) spectra were recorded on a Perkin LS-55 fluorescence spectrometer excited at 450 nm. Top-view and cross-sectional scanning electron microscope (SEM) images were obtained with a field-emission scanning electron microscope (Zeiss GeminiSEM 300, Germany). Transmission electron microscopy (TEM) images were obtained with a high-resolution transmission electron microscope (JEM-F200, Japan).Grazing incident wide angle X-ray scattering (GIWAXS) measurements were carried out on beamline BL14B1 at the Shanghai Synchrotron Radiation Facility (SSRF). FTIR spectra were recorded on a Nicolet iN10 spectrometer by mixing perovskite powder into potassium bromide (KBr). The time-resolved PL spectra were measured using a NanoLog Horiba Instruments spectrofluorimeter equipped with the integrating sphere. The X-ray photoelectron spectroscopy (XPS) tests were carried out with a photoelectron spectrometer model of ESCA LAB 250Xi from Thermo Fisher Scientific in the United States. Take A1 (Kα) (monochromatic aluminum Kα anode target, 1486.6 eV, the power is 150 W (15 kV, 10 mA)) as X-ray source, sampling depth: 1~10 nm, and the scanned area is 500 × 500 μm^2^. The X-ray exposure of the sample was only 15 min during the measurement. The charging correction was carried out with adventitious carbon at 284.8 eV. Atomic force microscopy (AFM), Kelvin probe force microscopy (KPFM), and conductive atomic force microscopy (c-AFM) measurements were performed using AFM microscope (Cypher ES, Oxford Instrument) with the AFM probe of Ti/Ir-coated Si cantilevers. Nano-infrared microscopy measurement was obtained by Anasys nanoIR3, Bruker. All the DFT calculations are performed using the Gaussian 16W software package. The geometry optimization and frequency calculation are carried out using the B3LYP functional with the 6-311+G(d,p) basis and Grimme's dispersion GD3 set.

*Device Testing: J-V* curves of the as-fabricated PSCs with different scanning directions were measured using a 2400 Sourcemeter (Keithley, USA) under simulated 1-sun AM 1.5G 100 mW cm^-2^ intensity (Oriel Sol3A Class AAA, Newport, USA). The typical active area of PSCs is 0.09 cm^2^ defined by a metal mask. The intensity of the 1-sun AM 1.5G illumination was calibrated using a Si-reference cell certified by the National Renewable Energy Laboratory. The External Quantum Efficiency (EQE) measurement was calculated using certified incident photon to current conversion efficiency equipment from Enlitech (Taiwan). The operational stability of PSCs was tested under continuous illumination with maximum power point tracking (20 ℃ in N_2_ flow). The light source is white LEDs and the light intensity was calibrated using a Si-reference cell. *J-V* curves were recorded every 6 h during the whole test.

*Trap density of state (tDOS) measurements:* The demarcation energy *E*_ω_ correlates with the applied frequency by the following formula: $\boldsymbol{E}_{\boldsymbol{\omega}}\boldsymbol{=}\text{k}_{\text{B}}\text{T}\text{ln}\text{(}\frac{\text{ω}_{\text{0}}}{\text{ω}}\text{)}$ (Equation 1)

where *ω_0_* is the attempt-to-escape frequency, *ω* is the applied angular frequency, *k_B_* is the Boltzmann constant, and *T* is the absolute temperature. And the distribution of trap density of states *N_T_* can be calculated by: ${\text{N}_{\text{T}}\boldsymbol{(E}}_{\boldsymbol{\omega}}\text{) }\boldsymbol{=}\text{- }\frac{\text{V}_{\text{bi}}}{\text{qW}} \frac{\text{dC}}{\text{dω}}\text{ }\frac{\text{ω}}{\text{k}_{\text{B}}\text{T}}\text{)}$ (Equation 2)

where *V_bi_* is the build-in potential, *W* is the width of the depletion region, and the thickness of active layer is employed as *W*. *q* is the elementary charge.

S-Q limit of *V*_OC_ and FF analysis: The maximum FF (FF_max_) can be empirically calculated by the following equation usually with the neglection of charge transfer loss:^1^ $\text{FF}_{\text{max}}\text{ }\text{= }\frac{\text{v}_{\text{oc}} \text{-}\text{ }\text{ln(}\text{v}_{\text{oc}}\text{ }\text{+}\text{ }\text{0.72)}}{\text{v}_{\text{oc}}\text{ }\text{+}\text{ }\text{1}}$ (Equation 3)

where $\text{v}_{\text{oc}}\boldsymbol{=}\frac{\text{q}\text{V}_{\text{oc}}}{\text{n}\text{k}_{\text{B}}\text{T}}$ (*q* is the elementary charge, *n* is the carrier density, *k*_B_ is the Boltzmann constant, and *T* is Kelvin temperature).

The S-Q limit of *V*_OC_ analysis is based on the reference.^2^

*Energy loss analysis:* The energy loss calculation follows reported work.^3-5^ In PSCs, the conversion of photons to electrons process occurs in distinct steps, which includes light absorption or exciton generation, exciton separation or free carrier generation, and charge extraction, each whit its own induced energy loss. The *transmission loss* (*E*_trans_), *insufficient light absorbing loss* (*E*_abs_), *thermalization energy loss* (*E*_thermal_), *V*_oc_ *loss* ($\text{E}_{\text{V}_{\text{oc}}}$), *IQE loss* (*E*_IQE_), *FF loss* (*E*_FF_), and *total converted energy* (*E*_conv_) are calculated by the following formulas:

(1) *Transmission loss* can be calculated by

$\text{E}_{\text{trans}}\text{ = }\int_{\text{0}}^{\text{E}_{\text{g}}} \text{Φ}\text{(}\text{hv}\text{)d}\text{hv}$ (Equation 4)

where *Φ*(*hv*) is the solar energy spectrum, *E*_g_ is the band gap of the solar cell system, and *hv* is the photon energy.

(2) *Insufficient light absorbing loss* can be calculated by

$\text{E}_{\text{abs}}\text{ = }\int_{\text{E}_{\text{g}}}^{\text{4.43}} \text{(1-EQE(100\% IQE, }\text{hv}\text{)}\text{Φ}\text{(}\text{hv}\text{))d}\text{hv}$ (Equation 5)

where the EQE (100% IQE, *hv*) is the EQE of the device assuming that the device IQE is 100%, which can be estimated from the optical simulation and reflectance spectra.

(3) *Thermalization energy loss* can be calculated by

$\text{E}_{\text{therm}}\text{ =} \int_{\text{E}_{\text{g}}}^{\text{4.43}} \left( \text{1-}\frac{\text{E}_{\text{g}}}{\text{hv}} \right)\text{EQE(}\text{hv}\text{)}\text{Φ}\text{(}\text{hv}\text{)d}\text{hv}$ (Equation 6)

(4) *V*_oc_ *loss* can be calculated by

$\text{E}_{\text{V}_{\text{oc}}}\text{ =} \int_{\text{E}_{\text{g}}}^{\text{4.43}} \left[ \frac{\left( \text{E}_{\text{g}}\boldsymbol{-}\text{V}_{\text{oc}} \right)}{\text{hv}} \right]\text{EQE(}\text{hv}\text{)}\text{Φ}\text{(}\text{hv}\text{)d}\text{hv}$ (Equation 7)

(5) *IQE loss* can be calculated by

$\text{E}_{\text{IQE}}\text{ =} \int_{\text{E}_{\text{g}}}^{\text{4.43}} \text{(1-IQE(}\text{hv}\text{))EQE(100\% IQE, }\text{hv}\text{)}\text{Φ}\text{(}\text{hv}\text{)d}\text{hv}$ (Equation 8)

(6) *FF loss* can be calculated by

$\text{E}_{\text{FF}}\text{ = (1-FF)}\left( \int_{\text{E}_{\text{g}}}^{\text{4.43}} \text{Φ}\text{(}\text{hv}\text{)d}\text{hv-}\text{E}_{\text{trans}}\text{-}\text{E}_{\text{abs}}\text{-}\text{E}_{\text{therm}}\text{-}\text{E}_{\text{V}_{\text{oc}}}\text{-}\text{E}_{\text{IQE}} \right)$ (Equation 9)

(7) Combining *E*_abs_ and *E*_IQE_

$\text{E}_{\text{abs}}\text{ +} \text{E}_{\text{IQE}}\text{ =}$ $\int_{\text{E}_{\text{g}}}^{\text{4.43}} \text{(1-EQE(}\text{hv}\text{)}\text{Φ}\text{(}\text{hv}\text{))d}\text{hv}$ (Equation 10)

Results and Discussion


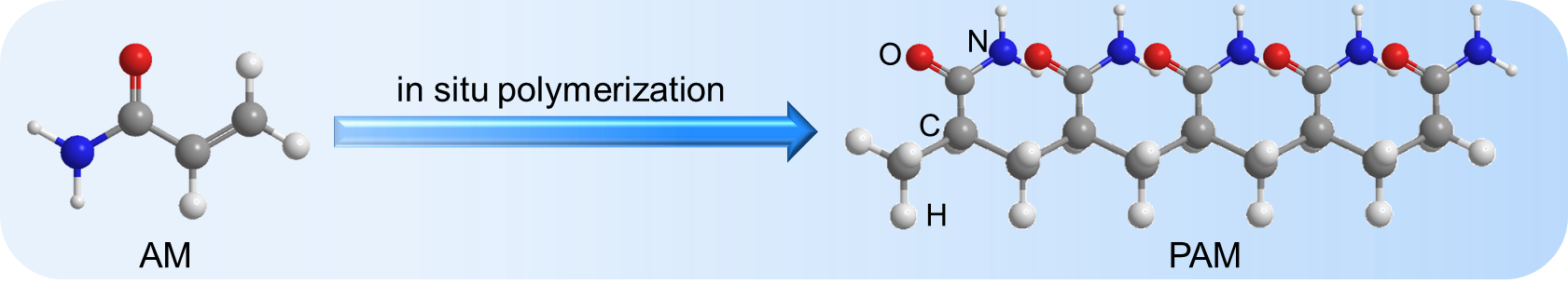


**Figure S1** Schematic diagram of in-situ polymerization reaction.

**Figure S2** XRD patterns of the control, PAM-, PAA-, PAN-, and PMA-treated perovskite films.

**Figure S3** XPS data of the control and PAM-treated perovskite films.

**Figure S4** Pb 4f and I 3d XPS spectra of the control and PAM-based films, respectively.

**Figure S5** (a) UV-vis absorption and (b) steady-state PL spectra of the control and PAM-based perovskite films.

**Figure S6** Time-resolved PL spectra of the control and PAM-based perovskite films.


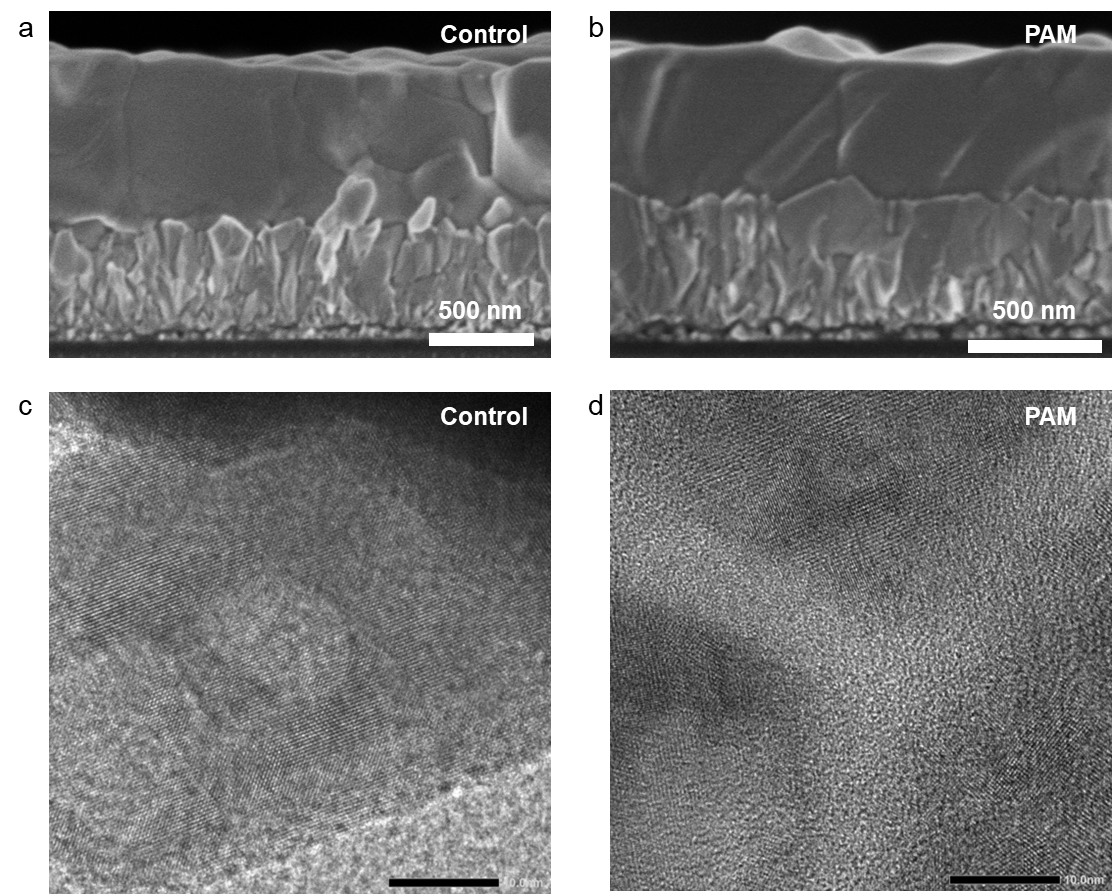


**Figure S7** Cross-sectional SEM and TEM images of (a, c) control and (b, d) PAM-based perovskite films.

**Figure S8** GIWAXS patterns of (a) control and (b) PAM-based perovskite films.

**Figure S9** In-situ PL spectra (a) without and (b) with AM monomer.

**Figure S10** Electrostatic potential profiles of monomer and pentamer.

**Figure S11** Theoretical interaction between PbI_2_ and monomer/pentamer.

**Figure S12** Binding energy between perovskite and monomer/pentamer.

**Figure S13** AFM morphology of (a) control and (b) PAM film with the scan size of 5 × 5 µm.

**Figure S14** (a, c) AFM morphology images and (b, d) Nano-IR images of control film and PAM-treated film. (The wavenumber of control film for nano-IR image is 1713 cm^-1^, and the wavenumber of PAM-treated film is 1675 cm^-1^.)

**Figure S15** *J*-*V* curves of PAM-based solar cells with different concentrations of AM additive.

**Figure S16** Certified efficiency with a designed area of 0.08713 cm^2^, confirmed by the Photovoltaic and Wind Power Systems Quality Test Center, IEE, Chinese Academy of Sciences, Beijing, P. R. China. No. PWQC-WT-P24013022-2R. The area of the mask was certified by National Institute of Metrology, China, No. CDjc2021-10891.

**Figure S17** EQE-*J*_SC_ curves of control and PAM-based solar cells.

**Figure S18** The boxplots of photovoltaic performance for control and PAM-based solar cells. (a) *V*_OC_, (b) *J*_SC_, (c) FF, and (d) PCE, respectively.

**Figure S19** *V*_OC_ response under different light intensities.

**Figure S20** The dark current-voltage curves of control and PAM-based solar cells.

**Figure S21** TPV curves of control and PAM-based solar cells.


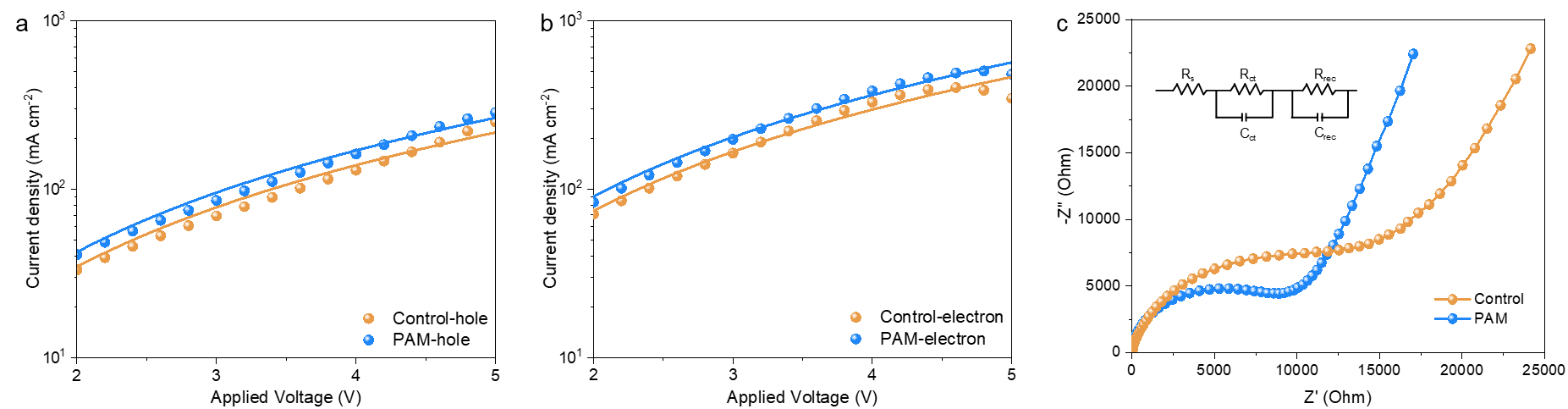


**Figure S22** Electrical characterization of the control and PAM-based solar cells measured in dark conditions. (a) Hole mobility plots. (b) Electron mobility plots.

(c) Nyquist plots.

**Figure S23** tDOS of the control and PAM-based solar cells.

**Figure S24** Schematic illustration of laser scribing by P1, P2, and P3 lines for PSC modules.

**Figure S25** (a) Schematic illustration and (b) SEM top-view image of the perovskite solar module showing the P1-P2-P3 parameters.

**Figure S26** Front-view of schematic diagram for perovskite solar module structure.

**Figure S27** The boxplots of photovoltaic performance for the control and PAM-based perovskite solar modules. (a) *V*_OC_, (b) *J*_SC_, (c) FF, and (d) PCE, respectively.

**Figure S28** S-Q limit of *V*_OC_ analysis.

**Figure S29** Detailed total energy loss analysis for the optimized: Control (a) and champion (b) device with the illumination of AM 1.5 G, 100 mW cm^-2^. (c) The corresponding Trans. Loss, Abs. and IQE Loss, *V*_OC_ loss, and FF loss.

**Figure S30** UV-vis absorption spectra with different heating time of (a) control and (b) PAM-treated perovskite film.

**Figure S31** XRD patterns of the control and PAM-treated perovskite film after aging for 503 h.

**Figure S32** Photo images of perovskite films for (a) control and (b) with PAM before and after aging.

**Figure S33** Top-view SEM images for control and PAM-based films after aging.

**Figure S34** Storage stability for the perovskite solar module.

**Table S1** Photovoltaic performance of PAM-based solar cells at different concentrations of AM.

| **Concentration (mg mL^-1^)** | ***V*_OC_ (V)** | ***J*_SC_ (mA cm^-2^)** | **FF (%)** | **PCE (%)** |
| --- | --- | --- | --- | --- |
| 0 | 1.126 | 25.29 | 79.99 | 22.78 |
| 2 | 1.151 | 25.44 | 81.48 | 23.86 |
| 4 | 1.168 | 25.33 | 82.21 | 24.32 |
| 6 | 1.173 | 25.49 | 83.40 | 24.93 |
| 8 | 1.211 | 25.63 | 83.88 | 26.05 |
| 10 | 1.196 | 25.32 | 82.70 | 25.05 |

**Table S2** Photovoltaic performance of control and PAM-based solar cells.

| **Device** | **Scan mode** | ***V*_OC_ (V)** | ***J*_SC_ (mA cm^-2^)** | **FF (%)** | **PCE (%)** | **HI (%) *^a^*** |
| --- | --- | --- | --- | --- | --- | --- |
| Control | Reverse | 1.157 | 25.43 | 79.64 | 23.43 | 4.0 |
|  | Forward | 1.133 | 25.27 | 78.67 | 22.48 |  |
| PAM | Reverse | 1.211 | 25.63 | 83.88 | 26.05 | 2.1 |
|  | Forward | 1.194 | 25.44 | 83.25 | 25.51 |  |

*^a^* Hysteresis index (HI) = (PCE_Reverse_ - PCE_Forward_)/PCE_Reverse_.

**Table S3** The analysis parameters of the total energy loss for the control and PAM-based solar cells.

| **Device** | ***E*_trans_** | ***E*_abs_ + *E*_IQE_** | ***E*_thermal_** | ***E*_Voc_** | ***E*_FF_** | ***E*_conv_** |
| --- | --- | --- | --- | --- | --- | --- |
|  | **(mW cm^-2^)** | **(mW cm^-2^)** | **(mW cm^-2^)** | **(mW cm^-2^)** | **(mW cm^-2^)** | **(mW cm^-2^)** |
| Control | 37.0 | 13.8 | 16.0 | 7.15 | 4.48 | 17.5 |
| PAM | 37.0 | 12.4 | 15.0 | 6.56 | 4.02 | 20.9 |

**Table S4** EDS elements analysis for the pristine films without and with PAM.

| **Film** | **Elements** | | | | **I/Pb ratio** |
| --- | --- | --- | --- | --- | --- |
|  | **C** | **N** | **I** | **Pb** |  |
| Control | 28.33 | 19.19 | 39.32 | 13.17 | 2.98 |
| PAM | 28.44 | 19.98 | 38.93 | 12.64 | 3.08 |

**Table S5** EDS elements analysis for the aged films without and with PAM.

| **Film** | **Elements** | | | | **I/Pb ratio** |
| --- | --- | --- | --- | --- | --- |
|  | **C** | **N** | **I** | **Pb** |  |
| Control | 20.22 | 4.91 | 51.80 | 23.07 | 2.24 |
| PAM | 21.10 | 11.82 | 47.89 | 19.19 | 2.50 |

**Author Contributions**

G. C., S. P., K. C., Z. S., and X. W. conceived and supervised the project. B. Z. fabricated and characterized the performance of the perovskite films and devices. Q. Z., K. G. and X. Z. assisted with the characterization of perovskite films and devices. C. G, X. S., and H. J. helped with the KPFM and device performance analysis. X. F. and Y. H. supported modules fabrication and characterization. G. C. commented on the results and provided constructive suggestions. All authors discussed the results and provided feedback on the manuscript. B. Z., X. W. and S. P. took the lead in drafting the manuscript and compiling the figures. G. C., K. C., S. P., Z. S., X. W. and B. Z. commented and reviewed the manuscript.

**References**

1. Wang, J.; Zhang, J.; Zhou, Y.; Liu, H.; Xue, Q.; Li, X.; Chueh, C.-C.; Yip, H.-L.; Zhu, Z.; Jen, A. K. Y., Highly efficient all-inorganic perovskite solar cells with suppressed non-radiative recombination by a Lewis base. Nat. Commun. 2020, 11 (1), 177.

2. Rühle, S., Tabulated values of the Shockley–Queisser limit for single junction solar cells. Solar Energy 2016, 130, 139-147.

3. Zuo, L.; Shi, X.; Fu, W.; Jen, A. K. Y., Highly Efficient Semitransparent Solar Cells with Selective Absorption and Tandem Architecture. Adv. Mater. 2019, 31 (36), 1901683.

4. Zuo, L.; Shi, X.; Jo, S. B.; Liu, Y.; Lin, F.; Jen, A. K. Y., Tackling Energy Loss for High-Efficiency Organic Solar Cells with Integrated Multiple Strategies. Adv. Mater. 2018, 30 (16), 1706816.

5. Cheng, J.; Cao, H.; Zhang, S.; Shao, J.; Yan, W.; Peng, C.; Yue, F.; Zhou, Z., Enhanced Electric Field Minimizing Quasi-Fermi Level Splitting Deficit for High-Performance Tin-Lead Perovskite Solar Cells. Adv. Mater. 2024, 36 (48), 2410298.

# Author Contributions

S.P.P. and X.W. and B.Q.Z. conceived and planned the experiments with additional input from G.L.C. and Z.P.S. and , B.Q.Z. fabricated all samples and devices, and performed and analyzed the SEM, the device efficiency experiments. X.F.D. performed the DFT calculation. D.C.L. and X.H.S. carried out the device stability test. Z.P.L. and L.Z.H. assisted with perovskite modules fabrication. C.Y.G. and Y.M.L assisted with film fabrication and XPS measurement. B.Q.Z. and X.W. and S.P.P. took the lead in drafting the manuscript and compiling the figures. All authors discussed the results and provided feedback on the manuscript.
